# Protein-polymer nano-machines. Towards synthetic control of biological processes

**DOI:** 10.1186/1477-3155-2-8

**Published:** 2004-09-06

**Authors:** Sivanand S Pennadam, Keith Firman, Cameron Alexander, Dariusz C Górecki

**Affiliations:** 1 School of Biological Sciences, Institute of Biomedical and Biomolecular Sciences, University of Portsmouth, Portsmouth PO1 2DT, UK; 2 School of Pharmacy and Biomedical Sciences, Institute of Biomedical and Biomolecular Sciences, University of Portsmouth, Portsmouth, PO1 2DT UK

## Abstract

The exploitation of nature's machinery at length scales below the dimensions of a cell is an exciting challenge for biologists, chemists and physicists, while advances in our understanding of these biological motifs are now providing an opportunity to develop real single molecule devices for technological applications. Single molecule studies are already well advanced and biological molecular motors are being used to guide the design of nano-scale machines. However, controlling the specific functions of these devices in biological systems under changing conditions is difficult. In this review we describe the principles underlying the development of a molecular motor with numerous potential applications in nanotechnology and the use of specific synthetic polymers as prototypic molecular switches for control of the motor function. The molecular motor is a derivative of a TypeI Restriction-Modification (R-M) enzyme and the synthetic polymer is drawn from the class of materials that exhibit a temperature-dependent phase transition.

The potential exploitation of single molecules as functional devices has been heralded as the dawn of new era in biotechnology and medicine. It is not surprising, therefore, that the efforts of numerous multidisciplinary teams [1,2]. have been focused in attempts to develop these systems. as machines capable of functioning at the low sub-micron and nanometre length-scales [3]. However, one of the obstacles for the practical application of single molecule devices is the lack of functional control methods in biological media, under changing conditions. In this review we describe the conceptual basis for a molecular motor (a derivative of a TypeI Restriction-Modification enzyme) with numerous potential applications in nanotechnology and the use of specific synthetic polymers as prototypic molecular switches for controlling the motor function [4].

## 1. Type I Restriction-Modification enzymes

**Figure 1:**
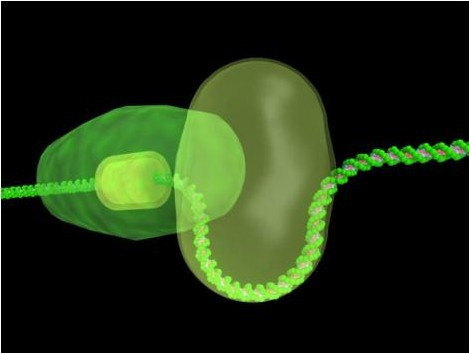
DNA Translocation by TypeI Restriction-Modification enzyme. The yellow block represents the recognition sequence for the enzyme. The enzyme binds at this site and upon addition of ATP, DNA translocation begins. During translocation, an expanding loop is produced.

Type I R-M enzymes are multifunctional, multisubunit enzymes that provide bacteria with protection against infection by DNA-based bacteriophage [5] They accomplish this through a complex restriction activity that cuts the DNA at random locations, which can be extremely distal (>20 kbp) from the enzyme's recognition sequence. In fact, the enzyme is capable of two opposing functions (restriction and modification), which are controlled enzymatically through an allosteric effector (ATP) and temporally through the assembly of the holoenzyme. In addition, the R-M enzyme has a powerful ATPase activity, which is associated with DNA translocation prior to cleavage; it is this translocation process that leads to random cleavage sites. Therefore, these enzymes are unusual molecular motors that bind specifically to DNA and then move the rest of the DNA through this bound complex (Fig 1).

**Figure 2:**
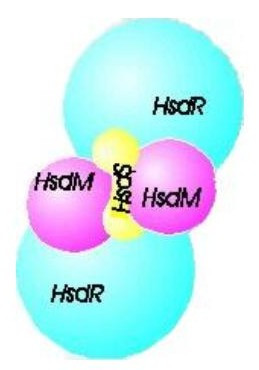
Schematic of the motor subunits. HsdS denotes the DNA binding subunit; HsdM – is the subunit responsible for DNA methylation and HsdR subunit, together with the core enzyme acts to restrict DNA.

Type I R-M enzymes fall into families based on complementation grouping, protein sequence similarities, gene order and related biochemical characteristics [6-8]. Within one sub-type (the IC family) there are three well-described members, including EcoR124I, which is the focus of our interest. This enzyme recognises the DNA sequence GAAnnnnnnRTCG [9] and is comprised of three subunits (HsdR,M,S) in a stoichiometric ratio of R_2_M_2_S [10,11], (Fig 2). However, Janscák *et al*. also showed that the *Eco*R124I R-M holoenzyme exists in equilibrium with a sub-assembly complex of stoichiometry R_1_M_2_S [11] which is unable to cleave DNA, but retains the ATPase and motor activity [12]. The HsdS subunit is responsible for DNA specificity; HsdM is required for DNA methylation (modification activity) and together they can produce an independent DNA methyltransferase (M_2_S) [13,14]. HsdR, along with the core MTase is absolutely required for DNA cleavage (restriction activity) and is also responsible for ATP-binding and subsequent DNA translocation. Therefore, the HsdR subunit is the motor subunit of the enzyme and this subunit is associated with helicase activity [15-18]. However, the precise mechanism of DNA translocation is uncertain and the true nature of the motor function has yet to be fully determined but a number of important functional units – nuclease, helicase and assembly domains have been identified within the HsdR subunit [19].

## 2. A versatile molecular motor

**Figure 3:**
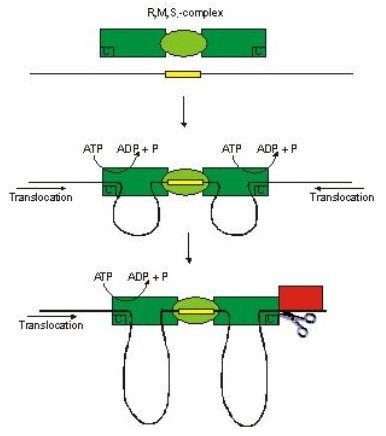
Mechanism of DNA cleavage. The enzyme subunits are represented by: green ellipse – M2S complex, green box – HsdR subunit (with ATPase and restrictase activities; C denoting cleavage site). The black line represents DNA with the yellow box denoting the recognition sequence. Arrow shows direction of DNA translocation. For more details see text.

The motor activity of Type I R-M enzymes is the mechanism through which random DNA cleavage is accomplished. Szczelkun *et al*. [20] showed that cleavage only occurs in a *cis *fashion indicating that the motor component of the HsdR subunit is able to 'grasp' adjacent DNA and pull this DNA through the enzyme-DNA-bound complex. According to the Studier model [21] cleavage occurs when two translocating enzymes collide (Fig 3). However, highly efficient cleavage of circular DNA carrying only a single recognition sites for the enzyme suggests collision-based cleavage is not the whole story [20,22].

**Figure 4:**
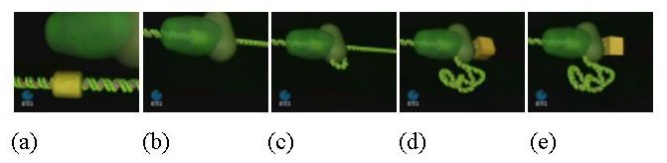
Motor activity of type I R-M Enzyme. (a) The yellow block represents the DNA-binding (recognition) site of the enzyme, which is represented by the green object approaching from the top of the diagram and about to dock onto the recognition sequence. (b) The motor is bound to the DNA at the recognition site and begins to attach to adjacent DNA sequences. (c) The motor begins to translocate the adjacent DNA sequences through the motor/DNA complex, which remains tightly bound to the recognition sequence. (d) Translocation produces an expanding loop of positively supercoiled DNA. The motor follows the helical thread of the DNA resulting in spinning of the DNA end (illustrated by the rotation of the yellow cube). (e) When translocation reaches the end of the linear DNA it stops, resets and then the process begins again.

DNA translocation has been assayed in bulk solution using protein-directed displacement of a DNA triplex and the kinetics of one-dimensional motion determined. The data shows processive DNA translocation followed by collision with the triplex and oligonucleotide displacement. A linear relationship between lag duration and inter-site distance gives a translocation velocity of 400 ± 32 bp/s at 20°C. Furthermore, this can only be explained by bi-directional translocation. An endonuclease with only one of the two HsdR subunits responsible for motion could still catalyse translocation. The reaction is less processive, but can 'reset' in either direction whenever the DNA is released (Fig 4).

As previously mentioned, the final step of the subunit assembly pathway of the Type I Restriction-Modification enzyme EcoR124I produces a weak endonuclease complex of stoichiometry R_2_M_2_S_1_. We have produced a hybrid HsdR subunit combining elements of the HsdR subunits of the EcoR124I and EcoprrI [23-25] Type I Restriction-Modification enzymes. This subunit has been shown to assemble with the EcoR124I DNA methyltransferase (MTase) to produce an active complex with low-level restriction activity. We have also assembled a hybrid REase and the data obtained show that the hybrid endonuclease (REase) containing only HsdR(prrI) is an extremely weak complex, producing primarily R_1_-complex. The availability of the hybrid REase produced from core MTase(R124I) and HsdR(prrI), which provides a stable R_1_-complex, also gives a useful molecular motor that will not cleave the DNA that it translocates.

## 3. Sub-cellular localisation of R-M enzymes

As can be seen from the above, DNA cleavage by Type I restriction enzymes occurs by means of a very unusual, and highly energy-dependent, mechanism. Therefore, these enzymes are believed to be involved not only as a defence mechanism for the bacterial cell, but also in some types of specialised recombination system controlling the flow of genes between bacterial strains [26,27]. A periplasmic location would be well adapted for the restriction activity of R-M enzymes, but recombination requires a cytoplasmic location. Restriction enzymes protect the cells by cutting foreign DNA and could be assumed to be located at the cell periphery. Using immunoblotting to analyse subcellular fractions, Holubova *et al. *[28] detected that the subunits of the R-M enzyme were predominantly in the spheroplast extract. The HsdR and HsdM subunits were found in the membrane fraction only when co-produced with HsdS and, therefore, part of a complex enzyme, either methylase or endonuclease. Further studies have shown that the R-M enzyme is bound to the membrane *via *the HsdS subunit and that for some enzymes this may involve DNA [29].

## 4. Uses of the EcoR124I molecular motor: polymer-protein conjugates in nanobiotechnology

One of the major obstacles for the practical application of single molecule devices is the absence of control methods in biological media, where substrates or energy sources (such as ATP) are ubiquitous. Synthetic polymers offer a robust and highly flexible means by which devices based on single biological molecules can be controlled. They can also be used to link individual biomacromolecules to surfaces, package them or to control their specific functions, thus expanding the applicability of the natural molecules outside conventional biological environments.

Moreover, a number of synthetic polymers have been recently developed that can potentially perform nanoscale operations in a manner identical to natural and engineered biopolymers. A key property of these materials is 'smart' behaviour, especially the ability to undergo conformational or phase changes in response to variations in temperature and/or pH. Synthetic polymers with these properties are being developed for applications ranging from microfluidic device formation, [30] through to pulsatile drug release [31-34], control of cell-surface interactions [35-39], as actuators [40] and, increasingly, as nanotechnology devices [41].

In the context of bio-nanotechnology we focus here on the uses of one particular subclass of smart materials, i.e. substituted polyacrylamides, but it should be noted that there are many more examples of synthetic polymers and engineered/modified biopolymers that exhibit responsive behaviour and new types and applications of smart materials are constantly being reported.

**Figure 5:**
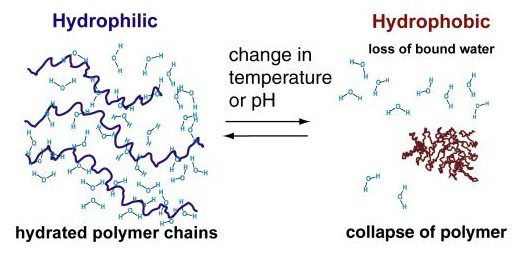
Inverse temperature solubility behavior of responsive polymers at the Lower Critical Solution Temperature (LCST). Left hand side shows hydrated polymer below LCST with entropic loss of water and chain collapse above LCST (right hand side).

Poly(N-isopropylacrylamide) (PNIPAm) is the prototypical smart polymer and is both readily available and of well-understood properties [42]. PNIPAm undergoes a sharp coil-globule transition in water at 32 °C, being hydrophilic below this temperature and hydrophobic above it. This temperature (the Lower Critical Solution Temperature or LCST) corresponds to the region in the phase diagram at which the enthalpic contribution of water hydrogen-bonded to the polymer chain becomes less than the entropic gain of the system as a whole and thus is largely dependent on the hydrogen-bonding capabilities of the constituent monomer units (Fig 5). Accordingly, the LCST of a given polymer can in principle be "tuned" as desired by variation in hydrophilic or hydrophobic co-monomer content.

### 4.1 Soluble PNIPAm-biopolymer conjugates

Covalent attachment of single or multiple responsive polymer chains to biopolymers offers the possibility of exerting control over their biological activity as, in theory at least, the properties of the resultant polymer-biopolymer conjugate should be a simple additive function of those of the individual components. This principle is now being widely exploited in pharmaceutical development, as covalent attachment of, for example, PEG chains to therapeutic proteins has been shown to stabilize the proteins without losing their biological function [43-48]. Polymer-biopolymer conjugates can be prepared as monodisperse single units, or as self-assembling ensembles depending on the chemistries used for attaching the synthetic component and on the associative properties of the polymer and/or biopolymer. Furthermore, by altering the response stimulus of the synthetic polymer, and how and where it is attached to the biopolymer, the activity of the overall conjugate can be very closely regulated. These chimeric systems can thus be considered as true molecular-scale devices.

Pioneering work in this area has been carried out by Hoffman, Stayton and co-workers, who engineered a mutant of cytochrome b5 such that a single cysteine introduced *via *site-directed mutagenesis was accessible for reaction with maleimide end-functionalised PNIPAm [49]. Since the native cytochrome b5 does not contain any cysteine residues this substitution provided a unique attachment point for the polymer. The resultant polymer-protein conjugate displayed LCST behaviour and could be reversibly precipitated from solution by variation in temperature. This approach has proved to be very versatile and a large number of polymer-biopolymer conjugates have now been prepared, incorporating biological components as diverse as antibodies, protein A, streptavidin, proteases and hydrolases [50,51,50,51]. The biological functions or activities of these conjugate systems were all similar to their native counterparts, but were switched on or off as a result of thermally induced polymer phase transitions. Of especial note have been the recent reports of a temperature and photochemically switchable endoglucanase, which displayed varying and opposite activities depending on whether temperature or UV/Vis illumination was used as the switch [52].

### 4.2. Controllable DNA packaging and compartmentalization devices

We are currently developing responsive polymers as a switch to control the EcoR124I motor function and are investigating this polymer-motor conjugate as part of an active drug delivery system. We aim for the practical demonstration of a nano-scale DNA packaging/separation and delivery system uniting the optimal features of both natural and synthetic molecules. In essence, we assemble a supramolecular device containing the molecular motor capable of binding and directionally translocating DNA through an impermeable barrier. To control the process of translocation in biological systems, where a constant supply of ATP is present, we have added to the motor subunit of EcoR124I the thermoresponsive poly(N-isopropylacrylamide) (PNIPAm), which, through its coil-globule transition, acts as a temperature-dependent switch controlling motor activity.

PNIPAm copolymers with reactive end-groups are being attached to a preformed R subunit of the motor *via *coupling of a maleimide-tipped linker on the synthetic polymer terminus to a cysteine residue. This residue has been selected, as it is both accessible and located close to the active centre on the R subunit of the motor. The protein-polymer conjugates are stable to extensive purification and, when combined with M2S complex, the activity of this conjugate motor system is similar to the native counterpart, but can be switched on or off as a result of thermally induced polymer phase transitions [53,54].

**Figure 6:**
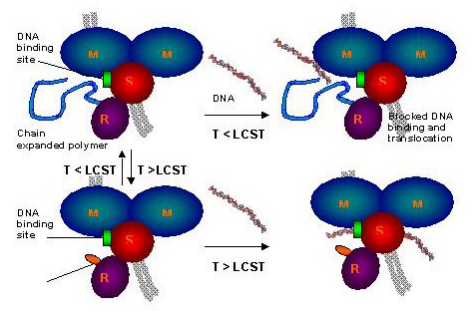
Schematic representation of the molecular motor function controlled by a thermoresponsive polymer switch. R, M and S denote the specific motor subunits. Chain-extension of the polymer below LCST provides a steric shield blocking the active site. Chain collapse (above LCST) enables access to the active site and restoration of enzyme function. For more details see text.

Thus the conjugation of the responsive polymer to the molecular motor generates a nano-scale, switchable device (Fig 6), which can translocate DNA under one set of conditions (i.e. into a protective capsule or into a compartment). Conversely, in another environment (e.g. inside cells), in response to changed conditions (e.g. changed temperature, pH) the polymer switch will change its conformation, allowing ATP to power the motor, releasing DNA from capsules or compartments.

The conjugation of the motor with synthetic polymers brings additional advantages. One such benefit arises from the ability to functionalise the polymer side chains or terminus in a way that allows attachment of the entire complex to surfaces for sensing and device applications.

Therefore, although our hybrid polymer-protein conjugate was originally aimed at gene targeting (as it has the potential to increase the delivery of intact DNA to cell nuclei and thereby increase gene expression) this system may also be used in building automated nano-chip sensors, therapeutic and diagnostic devices, where DNA itself would be a target, or where DNA might be used as a 'conveyor-belt' for attached molecules. The strength of the molecular motor has proven sufficient to disrupt most protein-DNA interactions and thus numerous processes and applications where highly localised force is required can also be envisaged.

## 5. Conclusions

The use of synthetic polymers offers a number of possibilities, which otherwise could not be exploited or would be difficult to take advantage of, if purely biological systems were used. Moreover, the combination of the properties of molecular motors with "smart" polymers has hitherto been unexplored and represents a novel concept in nanotechnology, which could ultimately lead to a wholly new class of molecular devices. Nanoscale control of molecular transport *in vitro *and especially *in vivo *opens up a whole host of possibilities in medicine, including drug or DNA delivery (e.g. gene therapy), but also where protection of a therapeutic is required under one biological regime and release in another (e.g. prodrugs conjugated to DNA which can be released by nuclease-mediated degradation at the site of action). In addition, this system may allow the generation of switchable nanodevices and actuators, controllable by changes in the synthetic copolymer structure as well as ATP-mediated DNA motion and may pave the way for biofeedback-responsive nanosystems. It can be used for nano-scale isolation of various biochemical processes in separate compartments connected *via *a tightly controlled shuttle device.

In essence, this concept bridges the disciplines of chemistry and biology by using a biological motor to control chemistry and a synthetic polymer to regulate biological processes.

## Author's contributions

KF conceived the idea of using the modified R-M enzyme as a molecular motor and carried out, with co-workers, the molecular studies of the motor components, SSP carried out the polymer synthesis, polymer-motor conjugations and functional studies, CA designed and participated in the synthesis of smart polymers and DCG conceived of the study. All authors participated in study design and coordination as well as the reading and approval of the final manuscript
